# Association of Vitamin D Genetic Risk Score with Noncommunicable Diseases: A Systematic Review

**DOI:** 10.3390/nu15184040

**Published:** 2023-09-18

**Authors:** Heba Almaghrbi, Mashael Al-Shafai, Maha Al-Asmakh, Hiba Bawadi

**Affiliations:** 1 Department of Biomedical Science, College of Health Sciences, QU Health, Qatar University, Doha P.O. Box 2713, Qatar; ha1707470@qu.edu.qa (H.A.); malshafai@qu.edu.qa (M.A.-S.); maha.alasmakh@qu.edu.qa (M.A.-A.); 2 Biomedical Research Center, Qatar University, Doha P.O. Box 2713, Qatar; 3 Department of Human Nutrition, College of Health Sciences, QU Health, Qatar University, Doha P.O. Box 2713, Qatar

**Keywords:** vitamin D, genetic risk score, noncommunicable diseases, single nucleotide polymorphisms, genetic predisposition, GWAS

## Abstract

**Background and Aims:** The genetic risk score (GRS) is an important tool for estimating the total genetic contribution or susceptibility to a certain outcome of interest in an individual, taking into account their genetic risk alleles. This study aims to systematically review the association between the GRS of low vitamin D with different noncommunicable diseases/markers. **Methods:** The article was first registered in PROSPERO CRD42023406929. PubMed and Embase were searched from the time of inception until March 2023 to capture all the literature related to the vitamin D genetic risk score (vD-GRS) in association with noncommunicable diseases. This was performed using comprehensive search terms including “Genetic Risk Score” OR “Genetics risk assessment” OR “Genome-wide risk score” AND “Vitamin D” OR 25(HO)D OR “25-hydroxyvitamin D”. **Results:** Eleven eligible studies were included in this study. Three studies reported a significant association between vD-GRS and metabolic parameters, including body fat percentage, body mass index, glycated hemoglobin, and fasting blood glucose. Moreover, colorectal cancer overall mortality and the risk of developing arterial fibrillation were also found to be associated with genetically deprived vitamin D levels. **Conclusions:** This systematic review highlights the genetic contribution of low-vitamin-D-risk single nucleotides polymorphisms (SNPs) as an accumulative factor associated with different non-communicable diseases/markers, including cancer mortality and the risk of developing obesity, type 2 diabetes, and cardiovascular diseases such as arterial fibrillation.

## 1. Introduction

The World Health Organization (WHO) defines noncommunicable diseases (NCDs) as chronic illnesses caused by a variety of genetic, physiological, environmental, and behavioral factors. These diseases are non-transmissible and non-infectious, and they primarily include cancer, obesity, diabetes and cardiovascular diseases [1]. NCDs pose a significant global health concern, accounting for 74% of global deaths and killing 41 million people annually. Cardiovascular diseases (CVDs) cause 17.9 million deaths, cancer causes 9.6 million deaths, obesity causes 4 million deaths, and diabetes causes 1.5 million deaths. By 2030, it is anticipated that the economic cost of chronic illnesses worldwide will reach USD 2.1 trillion [2]. Metabolic syndrome (MetS), characterized by elevated serum glucose, hypertension, and hyperlipidemia [3], is a significant risk factor for NCDs, including cancer [4], type 2 diabetes mellitus (T2DM) [5] and cardiovascular diseases [6]. The International Diabetes Federation (IDF) estimates that MetS affects one-quarter of the world’s population [7].

Vitamin D is a fat-soluble vitamin produced after the skin is exposed to sunlight, dietary intake, or supplementation. It is found in two forms, cholecalciferol (vitamin D3) and ergocalciferol (vitamin D2) [8]. Vitamin D_3_ has been reported to be crucial in regulating blood pressure and reducing hypertension [9,10]. However, other investigations have shown that vitamin D supplementation does not lower BP in the general population, and that the protective effect of vitamin D status against hypertension is unclear [11]. Interestingly, Vitamin D_3_ has also been claimed to significantly increase the overall survival outcome of cancer [12]. A study that underwent group analysis revealed a 12% lower cancer mortality rate in the vitamin D3 group compared with the placebo group in 10 trials with a daily dosing regimen [13]. Similar results were also reported by other studies, suggesting that the use of vitamin D supplementation in cancer patients could improve cancer survival [14,15]. Additionally, vitamin D_3_ has been reported to be crucial in reducing the risk of metabolic syndrome, including obesity [10], enhancing glucose uptake and improving insulin sensitization [16]. Vitamin D deficiency should be expected as an outcome of inadequate sunlight exposure, insufficient dietary vitamin D intake, malabsorption, and/or defective activation in the liver [17]. 

Various genetic factors, including genetic variations in the genes involved in vitamin D synthesis and metabolism, have been reported to influence vitamin D levels/deficiency status [18,19]. One such gene is 7-dehydrocholesterol reductase gene (*DHCR7*), which encodes the enzyme responsible for converting 7-dehydrocholesterol (7DHC) to cholesterol [20,21,22] and the 25-hydroxylase gene, cytochrome P450 2R1 (*CYP2R1*), which encodes the enzyme that converts vitamin D to its active circulating form 25(OH)D [21,23]. Vitamin D metabolism genes generally regulate the levels of biologically active 25(OH)D in the body. Cytochrome P450 24A1 (*CYP24A1*) helps to maintain the proper balance of blood 25(OH) concentrations by encoding the 24-hydroxylase enzyme that breaks down the active form of vitamin D to an inactive form [21,22,24]. The vitamin D-binding protein (*DBP*)/group-specific component gene (*GC*) is responsible for binding to vitamin D, and it serves as the transporter and reservoir for the principal vitamin D metabolites in the blood [25]. Another vitamin D-related gene is the vitamin D receptor gene (*VDR*), which encodes for a receptor for vitamin D, allowing the body to respond to vitamin D [26,27]. This also involves the calcium-sensing receptor gene (*CASR*), which has been shown to trigger the synthesis of 1,25(OH)D2 by regulating parathyroid hormone metabolism and calcium hemostasis [28,29]. 

Genetic predisposition is a known contributor to various NCDs [30,31]. This has been made evident by many genome-wide association studies (GWAS) that have revealed many single nucleotide polymorphisms (SNP) that are associated with a higher risk of several NCD diseases, including, but not limited to, metabolic diseases, cancers, and cardiovascular diseases [32,33]. 

The genetic risk score (GRS) is a genetic risk predictive model that can be constructed and calculated using an allelic scoring system that includes each of the risk alleles identified as being linked to the phenotype being studied [34]; it is an estimation of the total genetic contribution to a particular outcome of interest in an individual [35]. It could be calculated either as weighted, by taking into account the reported effect sizes for the selected alleles and hence adjusted for the total number of risk alleles and effect sizes evaluated, or unweighted, by simply counting the risk alleles and adding them as a GRS [35]. In this present study, we systematically review the association of the GRS of low-vitamin-D-risk alleles with different NCDs.

## 2. Methods

### 2.1. Registration of Protocol and Reporting

The International Prospective Registry of Systematic Reviews (PROSPERO CRD42023406929; https://www.crd.york.ac.uk/prospero/) received and approved a copy of the review protocol, and the PRISMA checklist was followed when reporting the review.

### 2.2. Literature Searches

The guidelines of the Preferred Reporting of Systematic Reviews and Meta-Analyses (PRISMA) statement served as the basis for the systematic review. The PubMed and Embase databases were searched from inception until March 2023. Only publications written in English were included. An extensive search regarding the vitamin D genetic risk score’s association with noncommunicable diseases was conducted. Search terms were based on all possible combinations of the following MeS terms and test keywords: “Genetic Risk Score” OR “Genetics risk assessment” OR “Genome-wide risk score” AND “Vitamin D” OR 25(HO)D OR “25-hydroxyvitamin D”. All retrieved articles went through initial screening and articles that fulfilled the inclusion criteria were included in this systematic review.

### 2.3. Eligibility Criteria

The used definition of NCDs is the one propounded by the WHO, which defines them as chronic diseases caused by a variety of genetic, physiological, environmental, and behavioral factors with four primary categories, including diabetes, cancer, chronic respiratory diseases, and cardiovascular disorders [36]. Hence, research papers were included in the review if they met the following inclusion criteria:

1. Studied the association between the calculated low vitamin D GRS and diabetes, cancer, chronic respiratory diseases, or cardiovascular disorders.
2. Calculated a genetic risk score (GRS) using selected low vitamin D level-related SNPs.
3. Measured at least one of the following NCD parameters (metabolic parameters such as blood lipids and fasting blood glucose (FBG), BMI, west circumference (WC), body fat percentage (BFP), insulin, or others like glucose, glycated hemoglobin (HbA1c), blood pressure, along with dietary intake analysis, etc.)

Articles were excluded if they:

1. Calculated the GRS with SNPs not related to vitamin D deficiency.
2. Studied the genetic predisposition to NCDs at the level of individual SNPs rather than collectively.
3. Studied SNPs that are associated with high vitamin D levels.
4. Were published as a review, book, protocol, guideline, or animal study.

### 2.4. Study Selection

**Figure 1:**
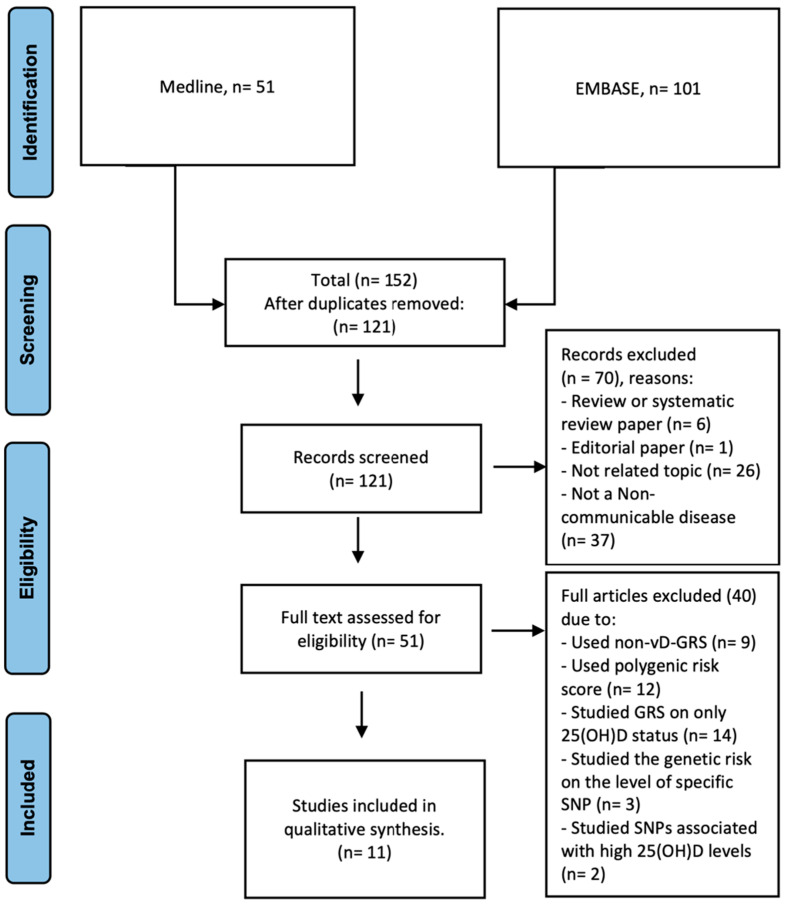
PRISMA flow-chart of the study selection process, 2022.

Records retrieved after the comprehensive search were assessed independently by two researchers (HB and HA), and any disagreements were resolved via consensus. Data screening and filtration were assisted by Rayyan (https://rayyan.ai/), an open-source website tool that assists researchers working on systematic reviews and other knowledge synthesis initiatives. The titles and abstracts of the articles were evaluated after removing duplicated articles and were assigned using Rayyan as included, excluded, or maybe. Any records that did not adhere to our inclusion criteria were eliminated. The entire full text was evaluated as part of the secondary selection process to resolve any “maybe” articles and gather pertinent data. When the two reviewers had differing views on an article that was classified as “maybe” at the final review, the article was discussed until a consensus was reached. A PRISMA flow chart illustrating the screening and selection procedure is shown in Figure 1.

### 2.5. Quality Control Assessment

Two researchers evaluated the quality of eligible articles for risk of bias using two different quality assessment tools. Cohorts, cross-sectional and case–control studies were evaluated using the Newcastle–Ottawa Scale (NOS) tool [37]. The Quality of Genetic Association Studies (Q-Genie) [38] tool was used to evaluate Mendelian randomization studies. When using the NOS tool, three groups of scoring criteria, totaling seven in all, were applied to the included cohort, cross-sectional and case–control studies. The study quality was determined by the studies’ selection of study groups, generalizability and verification of exposure and results, with few differences in the scoring parameters when adapted to different study designs; a total of eight points could be earned. Studies were deemed high-quality if they received more than seven points. Studies with 4–6 points were deemed to be of moderate quality, while those with 4 or fewer points were deemed to be of poor quality. However, using Q-Genie, we graded each screened article according to the following criteria using an 11-point scale [38]. Each point was scored from 1 (poor) to 7 (excellent). According to Q-Genie, studies without a control group with a score of ≤32 indicate poor quality, >32 and ≤40 indicate moderate quality, and >40 indicates high quality, which is applicable in our case since our Mendelian randomization studies have no control group. Any conflicts or disagreements were resolved via consensus.

### 2.6. Data Extraction

HA and HB collected the data independently for articles included in the systematic review and then discussed the final extraction. If any essential data needed to be understood, the study investigators of the relevant report were contacted. The variables extracted were divided into two sections: studies’ characteristics and the calculated genetic risk score description for each phenotype. For the studies’ characteristics, the following variables were extracted: (1) study design (i.e., cross-sectional, case–control, cohort, Mendelian randomization); (2) population studied (i.e., European, Asian, etc.); (3) population description (i.e., healthy or patients); (4) cohort name; (5) number of study participants; (6) age of study participants; (7) percentage of female participants; and (8) outcome phenotype studied (i.e., metabolic syndrome, cancer, cardiovascular diseases). Additional variables were extracted regarding the description of the vitamin D genetic risk score and its association. This includes (1) the number of SNPs used to calculate the VD_GRS; (2) the gene symbol and rs number of the SNPs selected by the study; (3) the reference study of the SNPs selected; (4) the VD_GRS computation method; (5) the VD_GRS construction criteria; (6) the association analysis model; (7) the non-genetic covariates the model was adjusted for; (8) the outcome indicators; and (9) the main significant findings. For each specific significant outcome finding, the *p*-value was presented to illustrate the significance of the included articles’ findings. 

## 3. Results

### 3.1. Search Outcome

A systematic search approach was employed for article screening and selection, following the PRISMA flow shown in Figure 1, which includes the results of the abstract and full-text screening and reasons for exclusion [39]. The initial literature search yielded 152 studies collected from Midline (*n* = 51) and Embase (*n* = 101), of which 121 remained after removing duplicates. After primarily screening titles and abstracts for eligibility, 70 records were excluded, leaving 51 research articles for full-text secondary screening. Finally, only 11 research articles were chosen after satisfying the inclusion and exclusion criteria [40,41,42,43,44,45,46,47,48,49,50]. These included five Mendelian randomization studies [42,43,44,46], two case–control studies [47,49], two cohort studies [48,50], and three cross-sectional studies [40,41,42]. Reasons for exclusion included the following: no calculation of vitamin D GRS, but the calculation of a polygenetic risk score instead (*n* = 12); GRS not based on vitamin D-related SNPs (*n* = 9); only serum vitamin D concentration studied as an outcome (*n* = 14); genetic predisposition of individual SNPs studied only (*n* = 3); and the use of SNPs associated with high 25(OH)D levels (*n* = 2).

### 3.2. Quality of the Eligible Studies 

The Newcastle–Ottawa Scale (NOS) tool [37] and Quality of genetic association studies (Q-Genie) tool [38] were utilized to perform the risk of bias assessment for the included observational studies and Mendelian randomization studies, respectively. The quality assessments of the included cross-sectional studies, cohorts, and case–control studies using the NOS tool in the three domains of selection, comparability and outcome are illustrated in Table 1, in sections A, B, and C, respectively. The cohort [48,50] studies and case–control [47,49] studies were given the quality scores “very good” and “good”, respectively, explaining their exceptionally high quality and illustrating little or no risk of bias. However, the included cross-sectional studies [40,41,45] were found to have a sort of bias in the selection domains, leaving them to be classified as “moderate” in terms of quality score. Since two of the included cross-sectional articles had no satisfactory (less than 400) sample size [41,45], no star was given in that sub-domain. Overall, the observational studies assessed the vD-GRS and its association with NCDs as high quality and could be considered reliable. 

On the other hand, all four Mendelian randomization studies [42,43,44,46] assessed for bias by the Q-Genie tool, Table 1 section D, were evaluated as “Good” since they had quality scores of more than 40 points. However, sources of bias were not fully discussed by most of the studies, especially when considering the gene–environment interaction our research question was asking, as it may be interpreted as an exclusion restriction violation for a Mendelian randomization study. Moreover, considering the details of the genotyping process is vital to a non-technical exposure classification, and most studies did not adequately describe how the genotyping was performed for all participants at once or in batches. Did a blinded assessor conduct the genotyping? These are essential aspects that are required to rate the domain according to the Q-Genie tool.

### 3.3. Characteristics of the Included Studies

Table 2 summarizes the key characteristics of the papers included in the systematic review. This review includes studies published between 2013 and 2022. Most articles were released between 2019 and 2022, with two additional papers published in 2017 and 2013. Mendelian Randomization studies were the most common in terms of study design (36.4%) [42,43,44,46], followed by cross-sectional studies (27.3%) [40,41,45]. Finally, case–control [47,49] and cohort studies [48,50] accounted for 18.2% of the studies. Out of the eleven studies included, five tested the genetic risk prediction score in individuals of Asian ancestry [40,42,44,45,47], another five tested that in individuals of European ancestry [43,46,48,49,50], and only one study focused on individuals of African ancestry [41]. Study sample sizes ranged from 110 [45] to 22,829 [49] for observational studies and from 699 [43] to 10,655 [42,44] for Mendelian randomization studies. The participants involved were either healthy or affected by the condition of interest (vitamin D deficiency). Furthermore, they were retrieved from different cohorts according to each study’s inclusion and exclusion criteria. The phenotypes studied included metabolic syndrome [40,42,43,45], T2DM [41], obesity [41], colorectal cancer [48,49,50], breast cancer [49], lung cancer [49], arterial fibrillation [47] and hypertension [48].

### 3.4. Genetic Risk Score Characteristics

The number of genetic variations assessed in the genetic risk score (GRS) prediction model varied between three [40] and eight [41] SNPs. A complete list of SNPs examined by each study is included in Table 3. The SNP most frequently included in the GRS calculation was rs2282679 of the DBP/GC gene, used in 11 out of 12 studies (91%). This was followed by rs6013897 of *CYP24AI*, used in 9 out of 12 studies (75%). Additionally, rs12785878 of *DHCR7* and rs10741657 of *CYP2R1* were each used in 8 out of 12 studies (66.6%). Other SNPs included in the GRS calculation were rs12794714 of *CYP2R1* and rs2228570 of *VDR*, which were each used in three studies (25%), while the variant rs1801725 of *CASR* was used in two studies (16%). The remaining six studies included different SNPs, each used only once in the GRS calculation. Most of the included studies obtained their intended SNPs from previously published genome-wide association studies (GWAS) or other published association studies that had shown a correlation with 25(OH)D concentrations. One study obtained SNPs from the HapMap project [50].

In contrast, another study stated that the SNPs used were known to be associated with 25(OH)D concentration, according to a previously published Mendelian randomization study of the Asian population [42]. Various approaches were examined in the studies that were considered to incorporate genetic factors into GRS prediction models. Of the twelve studies included, six used an unweighted simple count approach when constructing their GRS model. In contrast, the other six used a weighted additive model based on the total effect size. 

### 3.5. Vitamin D Genetic Risk Score Association with Metabolic Traits/Diseases

**Figure 2:**
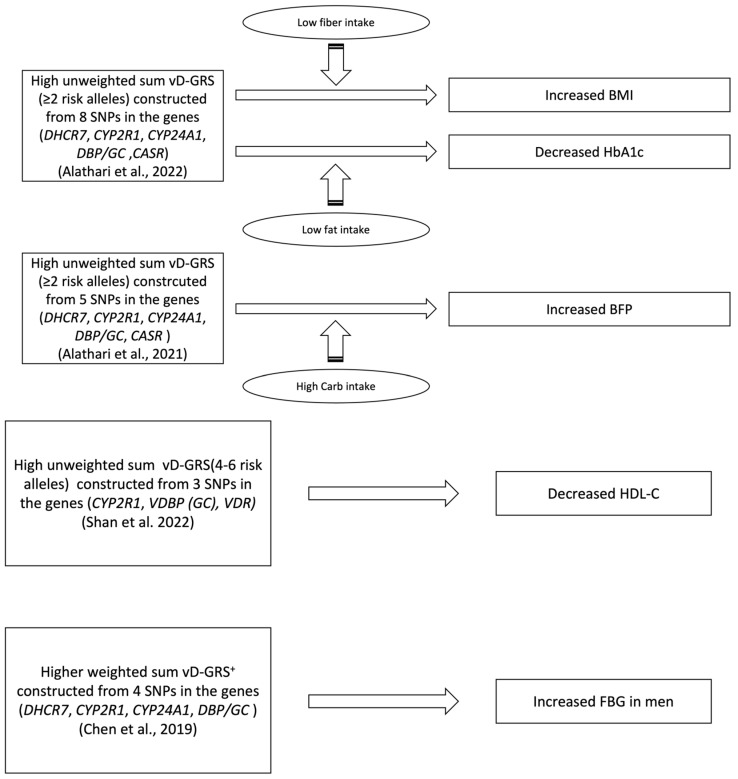
Significant associations found in the reviewed studies to study the association between vitamin D genetic risk score (vD-GRS) on metabolic traits. SNP: single nucleotide polymorphism; BMI: body mass index; HbA1c: glycated hemoglobin; BFP: body fat percentage; HDL-C: high-density lipoprotein- cholesterol; BFG, fasting blood glucose. + weighted vD-GRS was calculated in four quartiles; first quartile ≤2.480, second quartile 2.481–3.520; third quartile 3.521–4.640; fourth quartile ≥ 4.641 [40,41,42,45].

In this systematic review, six studies investigated the association between vitamin vD-GRS and metabolic traits [40,41,42,43,44,45]. A comprehensive brief description of the studies is shown in Table 4, section A and illustrated in Figure 2. In a cross-sectional study by Shan and Zhao [40], specific vitamin-D-metabolism-related SNPs and risk alleles were selected, including rs12794714 at cytochrome P450-2R1 (*CYP2R1*), rs2282679 at *VDBP (GC)*, and rs2228570 at vitamin D receptor (*VDR*), which were genotyped in Chinese participants and used to construct the vD-GRS. Metabolic parameters such as total cholesterol (TC), triglycerides (TG), low-density lipoprotein cholesterol (LDL-C), high-density lipoprotein cholesterol (HDL-C) and fasting blood glucose (FBG) were also measured for the participants. The results showed a significant association between the vD-GRS and both 25(OH)D concentrations and HDL-C levels, with a higher vD-GRS (4–6 risk alleles) being inversely associated with both (*p* value = 0.003 and <0.001, respectively). 

A second cross-sectional study conducted by Alathari and Nyakotey [41] investigated the association between vitamin-D-related genetic variants and dietary factors in obesity and T2DM as metabolic diseases in the Ghanaian population. The study selected eight SNPs associated with vitamin D concentration, and the GRS was calculated by summing the risk alleles across the eight SNPs. The SNPs included were rs2228570 and rs7975232 at *VDR*, rs12785878 at *DHCR7*, rs12794714 and rs10741657 at *CYP2R1*, rs6013897 at *CYP24A1*, rs2282679 at *DBP/GC*, and rs1801725 at *CASR*. The parameters measured in this study included glucose, glycated hemoglobin (HbA1c), insulin, TC, TG, and dietary intake analysis. The study found a significant correlation between the vD-GRS and fiber intake (g/day) concerning the body mass index (BMI) (P_interaction_ = 0.020). Specifically, individuals with a low fiber intake (≤16.19 g/day) and carrying more than two risk alleles for vitamin D deficiency had a significantly higher BMI (*p* = 0.01). Similarly, the study found an interaction between the vD-GRS and fat intake (g/day) on glycated hemoglobin (HbA1c), where individuals with a lower total fat intake (≤36.5 g/d) and carrying more than two risk alleles had significantly lower HbA1c levels (*p* = 0.049) (total fat, P_interaction_ = 0.029). 

Alathari and Aji [45] also conducted a nutrigenetic cross-sectional study on Indonesian women to explore the impact of vitamin-D-related genetic variants and dietary factors on metabolic-disease-related traits. The study used five SNPs in different genes associated with vitamin D synthesis and metabolism to calculate the GRS based on the number of risk alleles. The SNPs used were *DHCR7* rs12785878, *CYP2R1* rs12794714, *CYP24A1* rs6013897, *DBP/GC* rs2282679, and *CASR* rs1801725. The metabolic parameters measured were BMI, west circumference (WC), body fat percentage (BFP), glucose, HbA1c, insulin, TC, HDL-C, LDL-C, TG, and dietary intake analysis. The study found a significant interaction between the vD-GRS and carbohydrate intake in BFP (P interaction = 0.049), where individuals who consumed high amounts of carbohydrates and had more than two risk alleles had a significantly higher log BFP compared to those with fewer than or equal to two risk alleles (*p* = 0.016).

A mendelian randomization study conducted in China [42] investigated the effect of genetically lowered vitamin D levels on MetS and its related traits using a GRS constructed from four SNPs commonly associated with vitamin D, including rs12785878 in *DHCR7*, rs10741657 in *CYP2R1*, rs2282679 in *GC* and rs6013897 in *CYP24A1*. The study measured MetS parameters such as fasting plasma glucose (FPG), blood lipids, blood pressure or WC. The GRS was divided into three categories: GRS_combined_ (all included SNPs), GRS_synthesis_ (only *DHCR7* and *CYP2R1* SNPs), and GRS_metabolism_ (only *GC* and *CYP24A1* SNPs). The analysis revealed no correlation between the studied vD-GRSs and MetS components, except for a negative correlation with raised FPG in men (*p* = 0.003).

However, the last two Mendelian randomization studies of Lopez-Mayorga and Hauger [43], and Wang and Wang [44], which looked at the relationship between the vitamin-D-related SNPs’ genetic risk score (vD-GRS) and various cardiometabolic parameters, including FPG, HbA1c, BMI, TG, HDL-C, LDL-C and glycemic status, did not find any significant association between the genetic risk score and these markers. Lopez-Mayorga and Hauger [43] selected four low 25(HO) D-related SNPs, which were rs10741657 and rs10500804 at *CYP2R1* and rs4588 with rs12512631 in *GC*, and summed the total of risk alleles of rs10741657, rs10500804, rs4588 and rs12512631 to calculate their relative GRS. Meanwhile, Wang and Wang [44] used rs10741657 in *CYP2R1*, rs2282679 in *GC*, and rs601389 in *CYP24A1* 7 based on a previous GWAS study [51] to construct their weighted GRS upon its effect size with 25(OH)D obtained from extensive studies of Asian populations [52]. When each combined in a vD-GRS, both sets of SNPs showed no significant correlation with any metabolic markers except for a significant negative association with 25(OH)D serum concentration. 

### 3.6. Vitamin D Genetic Risk Score Association with Cancer

Among the three studies, as illustrated in Table 4, section B, that examined the effect of the vD-GRS on colorectal cancer (CRC) survival, mortality, and severity [48,49,50], only one prospective cohort study found limited evidence of a genetic predisposition towards low vitamin D levels having a direct impact on CRC mortality [48]. Neumeyer and Butterbach [48] used six vitamin-D-related SNPs to investigate the association between the vD-GRS and colorectal cancer (CRC) overall and specific survival in 7657 CRC patients who were followed up for a median of 54.8 months. The six SNPs were selected from GWAS studies [53,54] and included *GC* rs2282679, *CYP2R1* rs10741657, *DHCR7* rs12785878, *CYP24A1* rs6013897, *AMDHD1* rs10745742, and *SEC23A* rs8018720. The results showed that sex and BMI had a significant differential association with overall CRC mortality. Specifically, in women of a normal weight, a higher-weighted GRS was significantly associated with the increased overall mortality of CRC, indicating a heterogeneity effect. However, the study did not find any significant association between the calculated GRS and CRC-specific survival in general.

On the other hand, Yuan and Renfro [50] estimated the genetic risk predisposition of vitamin-D-deficiency-related SNPs in metastatic colorectal cancer. A prospective cohort of 535 CRC patients was genotyped for five vitamin-D-related SNPs, namely *GC* rs2282679, *CYP2R1* rs1993116, *DHCR7* rs12785878, *CYP24A1* rs6013897, and *NADSYN1* rs11234027, and the calculated GRS was tested for its association with overall survival, time to progression and tumor response. However, a Cox regression analysis revealed no association with any of the outcomes studied after a follow-up time of 9.2 years. Furthermore, an additional association analysis using the same GRS was conducted on breast and lung cancer incidence, which also revealed no significant association [50]. In a similar case–control study by Hiraki and Qu [49], 10,061 CRC cases were genotyped for four vitamin-D-related SNPs (*GC* rs2282679, *CYP2R1* rs10741657, *DHCR7/NADSYN1* rs12785878, and *CYP24A1* rs6013897) to examine the association between vD GRS and CRC risk, but no significant association was observed.

### 3.7. Vitamin D Genetic Risk Score Association with Cardiovascular Diseases

**Figure 3:**
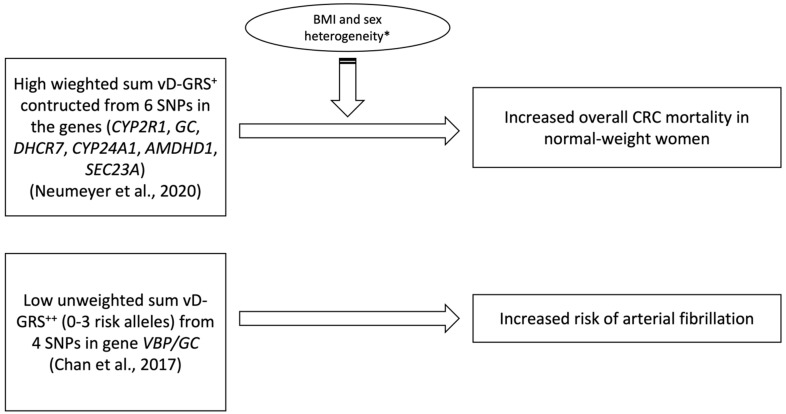
Significant associations found in included studies studying the effect of vitamin D genetic risk score (vD-GRS) on cancer and cardiovascular diseases. CRC, colorectal cancer. * Sex heterogeneity is when a correlation is limited to one gender other than the other. + The risk score ranges between 0 and 0.432. One unit change in GRS is associated to a change in vitamin D levels of 5.29 nmol/L [72]. ++ All risk-conferring alleles across the 4 SNP loci found associated with serum 25(OH)D were assigned a constituent score of 0, 1, or 2 based on allele frequency distribution [46,47].

Two studies in this review focused on the association between vD-GRS and cardiovascular diseases [46,47]. A comprehensive description of the studies is shown in Table 4, Section C and illustraited in Figure 3. In one case–control study that was conducted to examine the effect of vitamin-D-related SNPs on Arterial Fibrillation (AF), four SNPs at the vitamin D-binding protein (*VBP/GC*) gene were used, including rs4588, rs2282679, rs7041 and rs1155563 [47]. The study included 1019 controls and 156 cases, and interestingly, the GRS constructed by summing up the risk alleles of the four SNPs could strongly predict serum 25(OH)D (*p* < 0.001). A high GRS (4–8 summed risk alleles) predicted a genetically increased serum vitamin D status, while a low GRS (0–3) indicated a genetically deprived vitamin D status. More importantly, participants with a low GRS (0–3), indicating a genetically deficient vitamin D status, were at an elevated risk of AF compared to those with a high GRS (4–8) (*p* = 0.04), independent of other risk factors [47].

**Table 4:** Description of the vitamin D genetic risk score employed for the examination of the different phenotypes used.

First Author, Year [Ref]	Number of SNPs Selected	Gene Symbol (Rsnumber of SNPs Selected)	Reference Studies of SNPs	VD-GRS Computation	VD-GRS Construction Criteria	Association Model	Non-Genetic Covariates the Model Was Adjusted for	Outcome Indicators	Main Significant Findings
**Section A|Description of the vitamin D genetic risk score used for studies examining metabolic syndrome**									
Shan et al., 2022 [40]	3	CYP2R1 (rs12794714) GC (rs2282679) VDR (rs2228570)	Previous studies findings [55,56,57], GWAS, candidate SNPs	Unweighted, Simple Count	Summation of risk alleles of each SNP (range from 0 to 6 risk alleles	Linear regression model	Season, district, area type, latitude, age, BMI, PTH, P, ALT, CRE, IL-6, and hs-CRP	MetS’s Components (BMI, WC, SBP, DBP, TG, HDL-C, and FBG)	* ↑ vD-GRS w\ ↓ HDL-C (*p* = 0.003)
Alathari et al., 2022 [41]	8	DHCR7 (rs12785878) CYP2R1 (rs12794714, rs10741657) CYP24A1 (rs6013897) DBP/GC (rs2282679) CASR (rs1801725) VDR (rs2228570, rs7975232)	Previous studies findings [27,29,53,58,59,60,61,62,63,64], GWAS, candidate SNPs	Unweighted, Simple Count	Summation of risk alleles of each SNP (range from 0 to 6 risk alleles). Risk allele scores were then divided by the median of 2	General linear models with interaction analysis when needed	Age, gender, and BMI (when BMI was not an outcome), and total energy intake (only in the nutrient–GRS interaction analysis)	Biochemical and clinical metabolic outcomes (BMI and HbA1c) mediated or not by dietary intake	* ↑ vD-GRS x ↓ fiber intake (≤16.2 g/day) w\ ↑ BMI (Pinteraction = 0.020\ *p* = 0.010) * ↑ vD-GRS x ↓ fat intake (≤36.5 g/day) OR SFA w\ ↓ HbA1c (Pinteraction = 0.029\ *p* = 0.049) OR (Pinteraction = 0.044\ *p* = 0.049) respectively.
Alathari et al., 2021 [45]	5	DHCR7 (rs12785878) CYP2R1 (rs12794714) CYP24A1 (rs6013897) DBP/GC (rs2282679) CASR (rs1801725)	Previous studies findings [53,59,60,61,62,63,65,66,67], GWAS, candidate SNPs	Unweighted, Simple Count	Summation of risk alleles of each SNP (range from 0 to 6 risk alleles). Risk allele scores were then divided by the median of 2	Linear regression models with interaction analysis when needed	Age, BMI, location, and total energy intake.	Anthropometric and biochemical outcomes (BMI, WC, BFP, 25(OH)D, glucose, HbA1c, FBG, total cholesterol, HDL-c, LDL-c, and TG)	* ↑ vD-GRS x ↑ carbohydrate intake (mean ± SD: 319 g/d ± 46) w\ ↑ BFP (Pinteraction = 0.049\ *p* = 0.016)
Chen et al., 2019 [42]	4	DHCR7 (rs12785878) DBP/GC (rs2282679) CYP24A1 (rs6013897) CYP2R1 (rs1074165)	Mendelian Randomization study containing Asian participants [52]	Weighted, additive genetic model	Each SNP was coded 0–2 based on the number of effect alleles and then multiplied by the β value, followed by summing the four values.	Linear regression models and logistic regression	Age, sex, urban/rural residence, economic status, current smoking, WC, diabetes, hypertension, HDL-C	Biochemical and clinical metabolic outcomes (blood lipids, BP, FBG) Metabolic syndrome prevalence Anthropometric measurement (WC)	* ↑ VD-GRS w\ ↑ FBG (*p* = 0.003) for men
Wang et al., 2020 [44]	4	DHCR7 (rs12785878) DBP/GC (rs2282679) CYP24A1 (rs6013897) CYP2R1 (rs1074165)	Genome-wide association study on 25(OH)D [53]	Weighted, additive genetic model	Each SNP was coded 0–2 based on the number of effect alleles and weighted based on its effect size	linear and logistic regression analyses	Age, sex, urban/rural residence, economic status, current smoking, BMI, hypertension, HDL-C, LDL-C, TG and season variation (for	Biochemical and clinical metabolic outcomes (FPG, HbA1c, BMI, TG, HDL, LDL, Glycemic status, Type 2 diabetes, Prediabetes, Hypertension)	No association between the genetic risk score and any of the cardiometabolic markers.
Lopez-Mayorg et al., 2020 [43]	Mendelian randomization	GC (rs4588 and rs12512631) CYP2R1 (rs10741657 and rs10500804)	Previous studies from OPUS cohort and previous reported GWAS studies [53,68,69]	Unweighted, Simple Count	Three categories with increasing numbers of risk alleles (0–2, 3–5nd 6–8) calculated as the sum of the number of risk alleles	Linear and multiple linear regression models	Age, sex, parental education, entered puberty (yes/no), fat mass index and moderate-to-vigorous physical activity	Cardiometabolic markers (SBP, DBP, insulin, HDL-C, and TG)	No association between the genetic risk score and any of the cardiometabolic markers
**Section B|Description of the studies focusing on vitamin D genetic risk score in association with cancer**									
Neumeyer et al., 2020 [48]	6	GC (rs2282679) CYP2R1 (rs10741657) DHCR7 (rs12785878) CYP24A1 (rs6013897) AMDHD1 (rs10745742) SEC23A (rs8018720)	GWAS of European populations [53,54]	Weighted, additive genetic model	Sum of number of vitamin D decreasing alleles weighted based on its effect size	Cox proportional hazard models	Age, sex and principal components (PCs) of genetic ancestry	25(OH)D levels Overall survival Disease progression (time to progression) Tumor response	* ↑ vD-GRS w\ ↑ overall mortality in normal weight (*p* = 0.02) women (*p* = 0.01)
Yuan et al., 2020 [50]	5	GC (rs2282679) CYP2R1 (rs1993116) DHCR7 (rs12785878) CYP24A1 (rs6013897) NADSYN1 (rs11234027)	HapMap project	Weighted, additive genetic model	Each SNP was coded 0–2 based on the number of effect alleles and weighted based on its effect size	Cox proportional hazards regression	Age, sex, race/ethnicity, ECOG performance status, number of metastatic sites, and treatment arm	Cancer incidence Cancer Mortality Breast Cancer incidence Colorectal Cancer incidence Lung Cancer incidence Total incidence	No significant associations were found
Hiraki et al., 2013 [49]	4	GC (rs2282679) CYP2R1 (rs10741657) DHCR7/NADSYN1 (rs12785878) CYP24A1 (rs6013897)	GWAS of European populations	Unweighted, Simple Count	Summing the number of risk alleles yielding a possible range of 0-8 alleles	Generalized regression method [70]	Family history of CRC, BMI, NSAID use, alcohol use, dietary calcium, folate and red meat intake, sedentary status, and hormone replacement therapy	Colorectal Cancer Risk	No significant associations were found
**Section C|Description of the studies focusing on vitamin D genetic risk score in association with cardiovascular diseases**									
Chan et al., 2017 [47]	4	VBP/GC (rs4588 rs2282679, rs7041, rs1155563	Prior GWAS	Unweighted, Simple Count	linear continuous 0–8 constructed based on the summation method	Univariable and multivariable logistic regression	Age, gender, BMI, smoking, hypertension, diabetes mellitus, systolic/diastolic BP, triglycerides, LDL/HDL-c, creatinine, use of lipid-lowering drugs, and seasonal variation of recruitment	BMI, diabetes, renal function, diastolic BP and LDL-C, beta-blockers, angiotensin-converting enzyme inhibitors/angio-tensin receptor blockers, and lipid-lowering drugs	* ↑ vD-GRS w\ ↓ risk of arterial fibrillation
Magnus et al., 2018 [46]	4	DHCR7 (rs12785878) DBP/GC (rs2282679) CYP24A1 (rs6013897) CYP2R1 (rs1074165)	Prior GWAS studies [53,71]	Weighted, additive genetic model	Sum of number of vitamin D decreasing alleles weighted based on its effect size	Multivariable regression analysis	Gestational week of blood sampling and seven principal components to account for population stratification	hypertension gestational hypertension or pre-eclampsia	No consistent evidence of any associations of the tested GRSs with gestational hypertension or pre-eclampsia

The asterisk is for significant relationship.

Another Mendelian randomization study conducted by Magnus and Miliku [46] used a GRS composed of four vitamin-D-deficiency-related SNPs, including *DHCR7* rs12785878, *DBP/GC* rs2282679, *CYP24A1* rs6013897 and *CYP2R1* rs1074165, as a genetic instrument with which to examine the causal effect of genetically determined vitamin D status on gestational hypertension or pre-eclampsia, but no significant associations were detected. 

## 4. Discussion

To the best of our knowledge, this is the first systematic review to synthesize the available evidence regarding the association between the vitamin D deficiency genetic risk score and metabolic diseases, cancer, and cardiovascular diseases. We retrieved eleven published papers representing eleven different vitamin D genetic risk scores and collectively containing seventeen different SNPs of ten vitamin-D-associated genes. These studies investigated the association between the vD-GRS and different non-communicable diseases and their metabolic and cardiometabolic markers, colorectal cancer, arterial fibrillation, gestational hypertension, and pre-eclampsia. 

The association between the vD-GRS and different pathological phenotypes needs to be better addressed in the literature. However, it is mainly linked to metabolic diseases including, but not limited to, T2DM, hyperlipidemia and obesity. Out of the six studies that examined the association between different vD-GRSs and metabolic traits, two cross-sectional studies found a significant association with BMI, HbA1c, and BFP, mediated by dietary factors including fiber, fat, and carbohydrate intake, respectively [41,44]. As a diagnostic marker for obesity, BMI is predicted to increase in individuals with a high fat intake (≥56.52 g/day) and high a GRS (≥2 risk alleles) of vitamin D deficiency. In the same context, and since HbA1c is considered a marker for T2DM, it is expected to increase in people with a low fiber intake (≤16.19 g/day) and a high GRS (≥2 risk alleles) of vitamin D deficiency [41]. Similarly, the body fat composite was also reported to be increased with a high carbohydrate intake (mean ± SD: 319 ± 46 g/d) and a high GRS (≥2 risk alleles) of vitamin D deficiency [45]. All of these are consistent with general dietary recommendations [73]. More importantly, such observations highly emphasize following a high-fiber, low-fat and low-carbohydrate diet for those with a genetic risk of vitamin D deficiency to avoid developing T2DM and obesity in the future. Individuals genetically predisposed to low vitamin D status have a high vD-GRS, with high-risk alleles of different SNPs. Still, all are associated with 25(OH)D concentrations, mainly *GC* and *DHCR7* SNPs [74]. Therefore, a high vD-GRS is directly and significantly related to low vitamin D concentrations [63,74,75]. 

A recent meta-analysis, which included 24,600 children and adults, reported a significant association between obesity and vitamin D deficiency with a *p*-value of <0.01 [76]. Furthermore, multiple studies have suggested that vitamin D deficiency could be involved in obesity pathogenicity. This can be explained by the proposed regulatory role of vitamin D in adipogenesis, where it has been discovered that 1,25(OH)2D prevents adipogenesis by preserving the WNT/–catenin pathway, which is typically downregulated during adipogenesis [77]. Another older study reported that adipogenesis is inhibited in the early stages of differentiation by 1,25(OH)2D in a dose-dependent manner, by inhibiting the expression of the master transcription factors CAAT/enhancer-binding protein alpha (C/EBP) and peroxisome proliferator-activated receptor gamma (PPAR) [78]. This might suggest that low vitamin D levels could contribute to obesity via their failure to inhibit adipogenesis.

Similarly, observational studies consisting of 21 cohorts have linked low vitamin D levels with a higher risk of developing T2DM [79]. In this context, vitamin D has also been suggested to regulate insulin secretion, sensitivity, and glucose metabolism. People with high vitamin D levels have been found to have significantly higher insulin sensitivity, lower HbA1c levels, and lower triglyceride (TG) levels than those with low serum vitamin D [80]. However, the exact mechanism has yet to be established. Still, it has been proposed that inherited gene polymorphisms in the genes related to the vitamin D-binding protein (DBP), vitamin D receptor (VDR), and vitamin D 1alpha-hydroxylase (CYP1alpha) are connected to the potential role of vitamin D deficiency in insulin resistance [81]. 

Moreover, another cross-sectional study also found a significant differential association between the studied vD-GRS and HDL-C in men, where a high vD-GRS was associated with low HDL-C [40]. This association infers an adverse relationship effect, since HDL-C is known to reverse cholesterol transport into arteries, being beneficial for harmful cholesterol clearing and the prevention of many cardiovascular and metabolic diseases [82]. If low vitamin D genetic predisposition is associated with low HDL-C, low vitamin D serum levels are also linked to low HDL-C levels. According to research on insulin-resistant individuals, low vitamin D serum concentrations are associated with lower HDL-C levels, causing HDL-C dyslipidemia [83]. Another study in postmenopausal women also found that vitamin D deficiency is associated with lower HDL-C levels and that vitamin D supplementation leads to increased HDL-C levels [84]. It is unknown precisely how a vitamin D deficiency could result in a decline in HDL-C levels. However, lipid metabolism regulation is one possible explanation [85]. The remaining three studies found no significant correlation between the vD-GRS and metabolic traits.

Considering the three studies that examined the correlation between the calculated vD-GRS and CRC, only one study found a conditional differential significant association between the vD-GRS and the overall mortality of CRC in women of a normal weight [48]. The study suggested a possible correlation between female gender, a high BMI, low vitamin D status, and a genetic predisposition predictive score associated with decreased-vitamin-D-status risk alleles and the overall mortality of CRC. This might be explained by the fact that overweight and obese persons have lower vitamin D levels than people of a normal weight [86], which might be related to vitamin D sequestration in adipose tissues or the dilution of ingested vitamin D. The literature reports strong evidence of an association between BMI and low Vitamin D levels, and an increased risk of CRC mortality. Many studies have reported that the burden of death in CRC patients with a high BMI is steadily increasing. A global analysis study stated that CRC deaths reached almost 85.88 thousand between 1999 and 2019, and that the number is increasing [87]. Increasing evidence suggests that obesity-related insulin/IGF signaling pathways may promote invasion and metastasis, three of the ten characteristics of cancer, by maintaining proliferative signaling, evading apoptosis (or fighting cell death), and sustaining proliferative signaling [88]. A high BMI could also induce angiogenesis and promote inflammation, leading to the prognosis of CRC [88]. 

Regarding vitamin D levels, 25(OH)D has multiple mechanisms via which it protects against tumors by playing a vital role in regulating the expression of thousands of genes. This includes antiproliferation, apoptosis, DNA repair, prostaglandin metabolism, angiogenesis and the inhibition of metastasis [89]. The genetic predisposition of low vitamin D levels could increase CRC mortality. 

Two of the reviewed studies examined the association between the vD-GRS and cardiovascular diseases. One did not find any significant correlation between the constructed vD-GRS and gestational hypertension and pre-eclampsia [46]. However, the other study revealed that a genetically deprived vitamin D status independently predicted an increased risk of arterial fibrillation [47]. Researchers have reported the importance of the regulatory endocrine function of vitamin D in suppressing the activity of the renin–angiotensin–aldosterone system (RAAS), a hormonal system that plays a role in regulating blood pressure and fluid balance, hence preventing and reducing cardiovascular events and all-cause mortality [90,91]. Another study stated that the inhibition of RAAS reduces the incidence of atrial fibrillation in different patient groups with coronary artery disease [92]. Thus, any vitamin D deficiency would increase the risk of developing arterial fibrillation. A dose-dependent meta-analysis suggested that serum vitamin D deficiency is associated with an increased risk of AF [93]. 

This study has some limitations, including the relatively small number of studies included and the heterogeneity among the included studies in terms of the design, selected SNPs and GRS calculation methods. 

## 5. Conclusions

In conclusion, this systematic review reveals several significant associations between genetically deprived vitamin D and various health outcomes. These associations include increased colorectal cancer overall mortality, a higher risk of arterial fibrillation, and a dietary factors-mediated relationship, with body fat percentage, body mass index, and glycated hemoglobin. Additionally, there is a conditional differential association with increased fasting blood glucose in women. However, it is essential to note that genetically deprived vitamin D showed no association with certain cardiometabolic markers in two of the studies. Furthermore, two other studies found no evidence of an association with cancer incidence or risk. Additionally, one study did not provide consistent evidence of any associations between the tested genetic risk scores and gestational hypertension or pre-eclampsia. Moving forward, some critical unsolved questions must be investigated further. Exploring the mechanisms behind the observed associations, studying the potential impact of vitamin D supplementation on these outcomes, and addressing any confounding variables that may influence the results are all possible issues. Future research should strive to replicate these findings in bigger and more varied groups in order to improve the generalizability of the findings. 

## Figures and Tables

**Table 1:** Quality assessment for studies included.

**Section A|Newcastle-Ottawa Quality Assessment Scale Criteria for Cross-Sectional Studies**												
Study	Selection			Comparability			Outcome				Final Quality score	
	Representativeness of the cases	Sample size	Non-included subjects	The potential confounders were investigated using subgroup analysis or multivariable analysis			Assessment of the outcome		Statistical test			
Shan et al., 2022 [40]	*	*	*	**			*		-		6—Moderate	
Alathari et al., 2022 [41]	*	-	*	**			*		*		6—Moderate	
Alathari et al., 2021 [45]	*	-	*	**			*		*		6—Moderate	
**Section B|Newcastle-Ottawa Quality Assessment Scale Criteria for Cohort Studies**												
study	Selection			Comparability			Outcome				Final Quality score	
	Representativeness of the exposed cohort	Comparability of cohorts based on the design or analysis	Ascertainment of exposure	Demonstration that outcome of interest was not present at start of study	Comparability of cohorts based on the design or analysis		Assessment of the outcome		Was follow up long enough for outcomes to occur?	Adequacy of follow up of cohorts		
Neumeyer et al., 2020 [48]	*	*	*	*	**		**		*	*	9—Very Good	
Yuan et al., 2020 [50]	*	*	*	*	**		**		*	*	9—Very Good	
**Section C|Newcastle-Ottawa Quality Assessment Scale Criteria for Case–Control Studies**												
study	Selection			Comparability			Exposure				Final Quality score	
	Is the case definition adequate?	Representativeness of the cases	Selection of controls	Definition of controls	Comparability of cases and controls on the basis of the design or analysis		Assessment of the exposure		Same method of ascertainment for cases and controls	Non-Response rate		
Hiraki et al., 2013 [49]	*	*	*	*	*		*		*	N\A	7—Good	
Chan et al., 2017 [47]	*	*	*	*	*		*		*	N\A	7—Good	
**Section D|Quality of Genetic Association Studies (Q-Genie) for Mendelian Randomization Studies**												
Study	Rationale for study	Selection and definition of outcome of interest	Selection and comparability of comparison groups	Technical classification of the exposure	Non-technical classification of the exposure	Other sources of bias	Sample size and power	A priori planning of analyses	Statistical methods and control for confounding	Testing of assumptions and inferences for genetic analyses	Appropriateness of inferences drawn from results	Final Quality score
Chen et al., 2019 [42]	5	5	N\A	4	5	2	6	6	5	3	6	48—Good
Wang et al., 2020 [44]	6	5	N\A	4	2	2	6	6	6	4	5	46—Good
Lopez-Mayorg et al., 2020 [43]	4	6	N\A	5	2	3	3	6	3	4	5	41—Good
Magnus et al., 2018 [46]	6	6	N\A	6	4	3	5	6	6	5	5	52—Good

Each asterisk (*) is representing a score that will be summed as the final quality score, one asterisk is one score, two asterisk is two scores. N\A = Not Applicable for the type of the study assessed.

**Table 2:** Main characteristics of studies included in this review.

First Author, Year [Ref]	Study Design	Study Population	Population Description	Cohort Name	Number of Study Participants	Age (y, Range)	% Female	Outcome Phenotype Studied
Shan et al., 2022 [40]	Cross-sectional	Chinese	Non-diabetic women of childbearing age	2015 Chinese Adult Chronic Disease and Nutrition Surveillance (CCDNS)	1505	18–44	100	Metabolic Syndrome
Alathari et al., 2022 [41]	Cross-sectional	West African Ghanaian	Healthy Adults	Genetics of Obesity and Nutrition in Ghana (GONG)	302	25–60	~58.28	Obesity and type 2 diabetes
Alathari et al., 2021 [45]	Cross-sectional	Southeast Asian Minangkabau Indonesian	Healthy	Minangkabau Indonesia Study on Nutrition and Genetics (MINANG)	110	25–60	100	Metabolic diseases
Chen et al., 2019 [42]	Mendelian Randomization	Eastern Chinese	General population with detailed metabolic profiles measured	Survey on Prevalence in East China for Metabolic Diseases and Risk Factors (SPECT-China)	10,655	18–93	60	Metabolic Syndrome
Wang et al., 2020 [44]	Mendelian Randomization	Eastern Chinese	General population with detailed metabolic profiles measured	Survey on Prevalence in East China for Metabolic Diseases and Risk Factors (SPECT-China)	10,655	18–93	60	Type 2 Diabetes Pre-diabetes
Lopez-Mayorg et al., 2020 [43]	Mendelian randomization	Danish	Healthy Schoolchildren	OPUS School Meal Study	699	8–11	47	Metabolic Syndrome
Neumeyer et al., 2020 [48]	Prospective Cohort	European populations	CRC patients	International Survival Analysis in Colorectal Cancer Consortium (ISACC)	7657	-	54.6	Colorectal Cancer Survival
Yuan et al., 2020 [50]	Prospective Cohort	European populations	Patients with previously untreated mCRC	North Central Cancer Treatment Group (NCCTG) trial N9741	535	-	-	Metastatic Colorectal Cancer Survival
Hiraki et al., 2013 [49]	Case–Control	European population	Controls without colorectal adenocarcinoma Cases with colorectal adenocarcinoma	13 studies within Genetics and Epidemiology of Colorectal Cancer Consortium (GECCO) and Colon Cancer Family Registry (CCFR)	A total of 10,061 cases and 12,768 controls	-	53	Breast Cancer Colorectal Cancer Lung Cancer
Chan et al., 2017 [47]	Case–Control	Chinese	Controls without AF and cases with AF	Chinese clinical cohort of cardiac outpatients with stable coronary artery disease.	A total of 156 cases and 1019 controls	60.4–79.2	Cases: 30 Control: 25	Atrial Fibrillation
Magnus et al., 2018 [46]	Mendelian Randomization	European population	Patients with gestational hypertension or pre-eclampsia	Avon Longitudinal Study of Parents and Children (ALSPAC) and the Generation R Study	7389	-	100	Pregnancy-related Hypertensive Disorders

**Table 3:** SNPs identified in each study.

Vitamin D-GRS SNPs																	
Studies/Gene (SNP)	DHCR7 (rs12785878)	CYP2R1 (rs12794714)	CYP2R1 (rs10741657)	CYP2R1 (rs10500804)	CYP2R1 (rs1993116)	CYP24A1 (rs6013897)	DBP/GC (rs2282679)	GC (rs7041)	GC (rs12512631)	GC (rs3755967)	GC (rs4588)	VDR (rs2228570)	VDR (rs7975232)	AMDHD1 (rs10745742)	SEC23A (rs8018720)	CASR (rs1801725)	NADSYN1 (rs11234027)
Shan et al., 2022 [40]		✔					✔					✔					
Alathari et al., 2022 [41]	✔	✔	✔			✔	✔					✔	✔			✔	
Alathari et al., 2021 [45]	✔	✔				✔	✔									✔	
Chen et al., 2019 [42]	✔		✔			✔	✔										
Wang et al., 2020 [44]	✔		✔			✔	✔										
Lopez-Mayorg et al., 2020 [43]			✔	✔					✔		✔						
Neumeyer et al., 2020 [48]	✔		✔			✔	✔							✔	✔		
Yuan et al., 2020 [50]	✔				✔	✔	✔										✔
Hiraki et al., 2013 [49]	✔		✔			✔	✔										
Chan et al., 2017 [47]							✔	✔		✔		✔					
Magnus et al., 2018 [46]	✔		✔			✔	✔										

The tick mark is to refer that this SNP is calculated in the GRS by the cited study.

## Data Availability

Not applicable. No data was used for the research described in the article.

## Abbreviations

VD-GRS	vitamin D genetic risk score.
GWAS	genome wide association studies.
SNP	single nucleotide polymorphism.
MetS	metabolic syndrome.
BMI	body mass index.
PTH	parathyroid hormone.
P	phosphorus.
ALT	alanine aminotransferase.
CRE	creatinine; IL-6: interleukin 6.
hs-CRP	*C*-reactive protein.
WC	waist circumference.
BP	blood pressure.
SBP	systolic blood pressure.
DBP	diastolic blood pressure.
TG	triglyceride.
HDL-C	high-density lipoprotein cholesterol.
FBG	fasting blood glucose.
HbA1c	glycated hemoglobin.
SFA	saturated fatty acids.
BFP	body fat percentage.
LDL-C	low-density lipoprotein cholesterol.
CRC	colorectal cancer.
NSAIDs	non-steroidal anti-inflammatory drugs.
